# Role of the autonomic nervous system in young, middle-aged, and older individuals with essential hypertension and sleep-related changes in neurocardiac regulation

**DOI:** 10.1038/s41598-023-49649-2

**Published:** 2023-12-18

**Authors:** Chia-Hsin Yeh, Chun-Yu Chen, Yu-En Kuo, Chieh-Wen Chen, Terry B. J. Kuo, Kuan-Liang Kuo, Hong-Ming Chen, Hsin-Yi Huang, Chang-Ming Chern, Cheryl C. H. Yang

**Affiliations:** 1 Institute of Brain Science, National Yang Ming Chiao Tung University, No. 155, Sec. 2, Li-Nong St., Beitou, Taipei, 11221 Taiwan; 2 Sleep Research Center, National Yang Ming Chiao Tung University, Taipei, Taiwan; 3 School of Speech Language Pathology and Audiology, Chung Shan Medical University, Taichung, Taiwan; 4 Division of General Neurology, Neurological Institute, Taipei Veterans General Hospital, Taipei, Taiwan; 5 Faculty of Medicine, National Yang Ming Chiao Tung University, Taipei, Taiwan; 6 Clinical Research Center, Taoyuan Psychiatric Center, Ministry of Health and Welfare, Taoyuan, Taiwan; 7 Brain Research Center, National Yang Ming Chiao Tung University, Taipei, Taiwan; 8 Center for Mind and Brain Medicine, Tsaotun Psychiatric Center, Ministry of Health and Welfare, Nantou, Taiwan; 9 Department of Family Medicine, Taipei City Hospital Renai Branch, Taipei, Taiwan; 10 Institute of Biomedical Informatics, National Yang Ming Chiao Tung University, Taipei, Taiwan; 11 Department of Neurology, En Chu Kong (ECK) Hospital, 399 Fu-Xing Road, Sanxia District, New Taipei City, 23702 Taiwan; 12 Department of Education and Research, Taipei City Hospital, Taipei, Taiwan; 13 Department of Psychiatry, Chang Gung Medical Foundation, Chiayi Chang Gung Memorial Hospital, Chiayi, Taiwan; 14 Department of Psychiatry, Chang Gung University, Taoyuan, Taiwan; 15 Information Management Office, Taipei Veterans General Hospital, Taipei, Taiwan; 16 Department of Health and Leisure Management, Yuanpei University of Medical Technology, Hsinchu, Taiwan

**Keywords:** Cardiology, Diseases, Neurology

## Abstract

Essential hypertension involves complex cardiovascular regulation. The autonomic nervous system function fluctuates throughout the sleep–wake cycle and changes with advancing age. However, the precise role of the autonomic nervous system in the development of hypertension during aging remains unclear. In this study, we characterized autonomic function during the sleep–wake cycle in different age groups with essential hypertension. This study included 97 men (53 with and 44 without hypertension) aged 30–79 years. They were stratified by age into young (< 40 years), middle-aged (40–59 years), and older (60–79 years) groups. Polysomnography and blood pressure data were recorded for 2 min before and during an hour-long nap. Autonomic function was assessed by measuring heart rate variability and blood pressure variability. Data were analyzed using *t* tests, correlation analyses, and two-way analysis of variance. During nonrapid eye movement (nREM), a main effect of age was observed on cardiac parasympathetic measures and baroreflex sensitivity (BRS), with the highest and lowest levels noted in the younger and older groups, respectively. The coefficients of the correlations between these measures and age were lower in patients with hypertension than in normotensive controls. The BRS of young patients with hypertension was similar to that of their middle-aged and older counterparts. However, cardiac sympathetic activity was significantly higher (*p* = 0.023) and BRS was significantly lower (*p* = 0.022) in the hypertension group than in the control group. During wakefulness, the results were similar although some of the above findings were absent. Autonomic imbalance, particularly impaired baroreflex, plays a more significant role in younger patients with hypertension. The nREM stage may be suitable for gaining insights into the relevant mechanisms.

## Introduction

Essential hypertension is the most common type of hypertension in adults, and its pathophysiology is complex and multifactorial^1^. The autonomic nervous system (ANS) regulates the cardiovascular system^2^, and autonomic dysfunction is one of the most important mechanisms in essential hypertension^3^. The etiopathology of autonomic dysfunction in hypertension includes chemoreceptor activation, insulin/leptin-mediated sympathetic activation, cardiopulmonary reflex dysfunction, sodium homeostasis disruption, the renin–angiotensin system overactivation, and other humoral systems problem^2^. Both animal and human studies have concluded that increased sympathetic activity and reduced vagal modulation are associated with hypertension^4,5^. Hypertension is an age-related disorder, and the interactions between its etiological factors changes with age^6^. However, few studies have comprehensively investigated the role of the ANS in the development of hypertension during the aging process.

With aging, sympathetic activity increases and parasympathetic activity decreases in normotensive individuals^7–9^. Studies have reported that irrespective of age, patients with hypertension exhibit weak autonomic responses to postural changes^10^ and enhanced sympathetic nerve traffic^2^. However, in a relevant study, although younger (< 60 years) and older (≥ 60 years) patients with hypertension had similar resting heart rates, the older patients exhibited reductions in baseline cardiac sympathetic and parasympathetic tones^11^. According to the available studies, no definitive conclusions have been reached regarding age-dependent alterations in autonomic function among individuals with hypertension.

One possible reason may be that most studies involving patients with hypertension have assessed autonomic function exclusively during wakefulness. Nevertheless, autonomic function significantly fluctuates during the sleep–wake cycle. Muscle sympathetic nerve activity, blood pressure (BP), heart rate, and arterial pressure variability decrease^12^ and baroreflex resetting and parasympathetic cardiac regulation increase^13,14^ during the nonrapid eye movement (nREM) stage compared with the data recorded during wakefulness. We previously reported that the sympathovagal balance in Wistar–Kyoto rats shifted toward vagal limb dominance during sleep; this shift was absent in spontaneously hypertensive rats, who exhibited strengthened sympathetic vasomotor activity but weakened baroreflex sensitivity (BRS) during sleep^15,16^.

The autonomic parameters derived from heart rate (HRV) and BP variability can facilitate the detection and treatment of hemodynamic and autonomic disturbances in patients with essential hypertension^17,18^. In this study, we investigated the changes in cardiovascular neural regulation using HRV and BP variability during both sleep and wakefulness in different age groups with essential hypertension. We hypothesized that autonomic function during the sleep–wake cycle and at various ages would vary between patients with hypertension and individuals without hypertension.

## Methods

### Participants

This study included 97 men aged 30–79 years. They were divided into the hypertension and control groups. The hypertension group comprised 53 men who were diagnosed as having essential hypertension; the diagnosis was made by physicians in accordance with the criteria outlined in the Seventh Report of the Joint National Committee on Prevention, Detection, Evaluation, and Treatment of High Blood Pressure. Additionally, secondary hypertension was excluded in accordance with its guidelines^19^. The control group comprised 44 age- and sex-matched men without hypertension (i.e., normal BP, defined as systolic/diastolic BP of < 140/90 mmHg). Based on research indicating sex-specific variations crucial for BP regulation within sympathetic neural-hemodynamic equilibrium, encompassing sympathetic nerve activity, cardiac output, and β-adrenergic responsiveness, this study conducts an in-depth examination specifically within males^20^.

Men with a history of kidney disease, adrenal disease, thyroid problems, obstructive sleep apnea, diabetes mellitus, arrhythmia, congestive heart failure, cardiovascular disease, cerebrovascular disease, malignancy, active infectious disease, or psychiatric illness were excluded from this study. No participant had substance abuse problems, alcohol addiction, or psychiatric illness. Written informed consent was obtained from all participants. The study protocol was approved by the institutional review boards of Taipei Veterans General Hospital and National Yang Ming Chiao Tung University (approval numbers: 96-08-15A and YM108144E, respectively).

### Experimental procedure and data recording

The participants were stratified by age into three groups: younger (30–39), middle-aged (40–59), and older (60–79) groups. This study was conducted at the Sleep Center of the Institute of Brain Science, National Yang Ming Chiao Tung University, Taipei, Taiwan. During the test period, the participants were instructed to nap in a sound-attenuated room (humidity: 55–60%; temperature: 24.40 °C ± 0.78 °C) and their polysomnography (PSG; Embla SX, Natus Medical Incorporated, USA) and BP data were recorded for 1 h. During PSG, central and occipital electroencephalography (EEG; C3–A2, C4–A1, O1–A2, and O2–A1), electrooculography (EOC-L and EOC-R), chin and anterior tibialis electromyography (chin and limb), and electrocardiography (ECG; V5 on the chest) were performed to evaluate the participants’ cardiac activity. Sleep stages were scored in congruence with the standard criteria in the literature^8,21,22^. BP was continuously measured using Finometer PRO (Finometer Model-1/Pro, FMS01; FMS, Amsterdam, the Netherlands). An upper-arm cuff was used for finger blood calibration, and a 2-min baseline recording was conducted before sleep. Synchronized integration of real-time BP signals was performed by the PSG software Somnologica (Embla, Denver, CO, USA).

### Signal processing and data analysis

A power spectral analysis was conducted to measure the frequency domain of HRV. The R-R interval (RR) signal was divided into continuous 64-s (4096 points) time segments (windows or epochs) with a 50% overlap. The circumstantial analytical procedures used in HRV analysis have been thoroughly discussed in previous studies^23–25^. Through Fourier transformation, the spectral information of HRV was sorted into total power (TP), high frequency (HF), and low frequency (LF). HF (0.15–0.4 Hz), LF (0.04–0.15 Hz) to HF ratio (LF/HF), and normalized LF (LF%) of the RR spectrogram were enumerated in each time segment. RR and TP are associated with both sympathetic and parasympathetic nerves^26^. HF reflects vagal modulation, whereas LF% and LF/HF ratio indicate sympathetic modulation or the sympathovagal balance^27^.

For the sleep stage analysis, computerized sleep analysis was conducted following the guidelines of Rechtschaffen and Kales^28^ and the American Academy of Sleep Medicine^8,29^. Later, the results were reconfirmed by a qualified sleep technician. All polysomnographic data were manually scored on a 30 s-epoch basis, from what we calculated wakefulness and nREM sleep.

To analyze the EEG and ECG data, we designed a special algorithm in Pascal language (Borland Pascal 7.0, Borland, USA). ECG signals were preprocessed as described previously^26,30^. In brief, using this algorithm, the ventricular premature complex and artifact were identified and the QRS complex was acquired. For the temporal continuity, the stationary RR signals were resampled and linearly interpolated at a rate of 64 Hz. The sampling rate was adjusted to 64 Hz also for the EEG signals.

EEG amplitudes, arterial pressure variability, and HRV were measured through power spectral analysis. The EEG, arterial pressure, and RR signals were incised into successive 64-s (4096 points) time segments (windows or epochs) with a 50% overlap. Fast Fourier transformation was used to estimate the power density of the spectral components, and the attenuation originating from sampling was corrected. A Hamming window was used for the aforementioned procedures^24,26^. The detailed evaluation of arterial pressure variability and HRV has been described previously^15,23,25–27^. In brief, mean arterial pressure derived from digitized arterial pressure signals and the mean RR were calculated from the digitized ECG signals. To assess temporal continuity, resampling and interpolating were performed. Fast Fourier transformation and a Hamming window were adopted for these sequences^24^. Both HF (0.15–0.4 Hz) and LF% (0.04–0.15 Hz) were gauged from the RR spectrogram. LF of blood pressure variability (BLF) (0.04–0.15 Hz) was quantified from the arterial pressure spectrogram for each time segment. BLF serves as an indicator of sympathetic vasomotor control^25,27^.

Spontaneous BRS was calculated from the arterial pressure–RR transfer function and arterial pressure–RR linear regression^25,27^. The transfer magnitude at HF (BrrHF) and LF (BrrLF) ranges were estimated using the transfer function analysis. For the linear regression analysis, the slopes of the arterial pressure–RR pair simultaneously ascended (BrrA) and descended (BrrD). Finally, the mean values of arterial pressure variability, HRV, and BRS were obtained during each sleep–wake cycle of each participant.

### Statistical analysis

Descriptive statistics are presented in terms of the mean ± standard deviation values. Independent-samples *t* tests were conducted to determine the differences between patients with hypertension and individuals without hypertension across the age groups. Pearson correlation coefficients were calculated to measure the associations between HRV, BP, arterial pressure variability, BRS parameters, and age. We are simultaneously conducting a comparison of correlations from independent samples^31^. Two-way analysis of variance (ANOVA), followed by a least-significant difference post hoc test, was performed to compare the variables across different age groups (30–39, 40–59, and 60–79 years) and different BP states. The analysis was performed considering the interaction effect. The significance level was set at 0.05. All analyses were conducted using SPSS (version 22; IBM, Armonk, NY, USA).

### Ethics approval and consent to participate

This study was fully reviewed and approved by the Ethics Institutional Review Board of Taipei Veterans General Hospital and National Yang Ming Chiao Tung University.

## Results

### Cohort characteristics

No significant difference was observed in the mean age between patients with hypertension and individuals without hypertension in any age group. Except for the increased body mass index (BMI) observed in middle-aged patients with hypertension (*p* = 0.005), no significant differences were noted in BMI between patients with hypertension and individuals without hypertension in any other age group (Table 1). In the younger group, patients with hypertension had significantly higher SBP than did individuals without hypertension (*p* = 0.001). In the middle-aged and older groups, patients with hypertension had significantly higher DBP than did individuals without hypertension (*p* = 0.001 and 0.010, respectively).

**Table 1:** Demographic characteristics of the study population.

Demographics	Individuals without hypertension			Patients with hypertension		
	Younger group	Middle-aged group	Older group	Younger group	Middle-aged group	Older group
Number	n = 14	n = 13	n = 17	n = 5	n = 16	n = 32
Age (year)	32.1 ± 2.1	50.3 ± 5.3	68.5 ± 4.2	33.8 ± 3.6	51.2 ± 6.2	70.1 ± 6.5
BMI (kg/m^2^)	22.5 ± 2.1	23.4 ± 1.0	23.0 ± 2.6	26.6 ± 4.4	26.3 ± 3.3*	24.1 ± 2.5
SBP (mmHg)	121.6 ± 8.1	126.9 ± 13.1	131.0 ± 14.7	143.8 ± 11.7*	136.1 ± 13.6	135.7 ± 15.3
DBP (mmHg)	72.6 ± 8.0	73.3 ± 3.2	78.8 ± 12.1	94.8 ± 12.7*	87.4 ± 12.4*	83.2 ± 8.4

Mean ± SD.

*BMI* body mass index, *SBP* systolic blood pressure, *DBP* diastolic blood pressure.

*p < 0.05 vs. control group, independent *t* test.

### Effects of age and hypertension on cardiac autonomic measures during sleep and wakefulness

**Figure 1:**
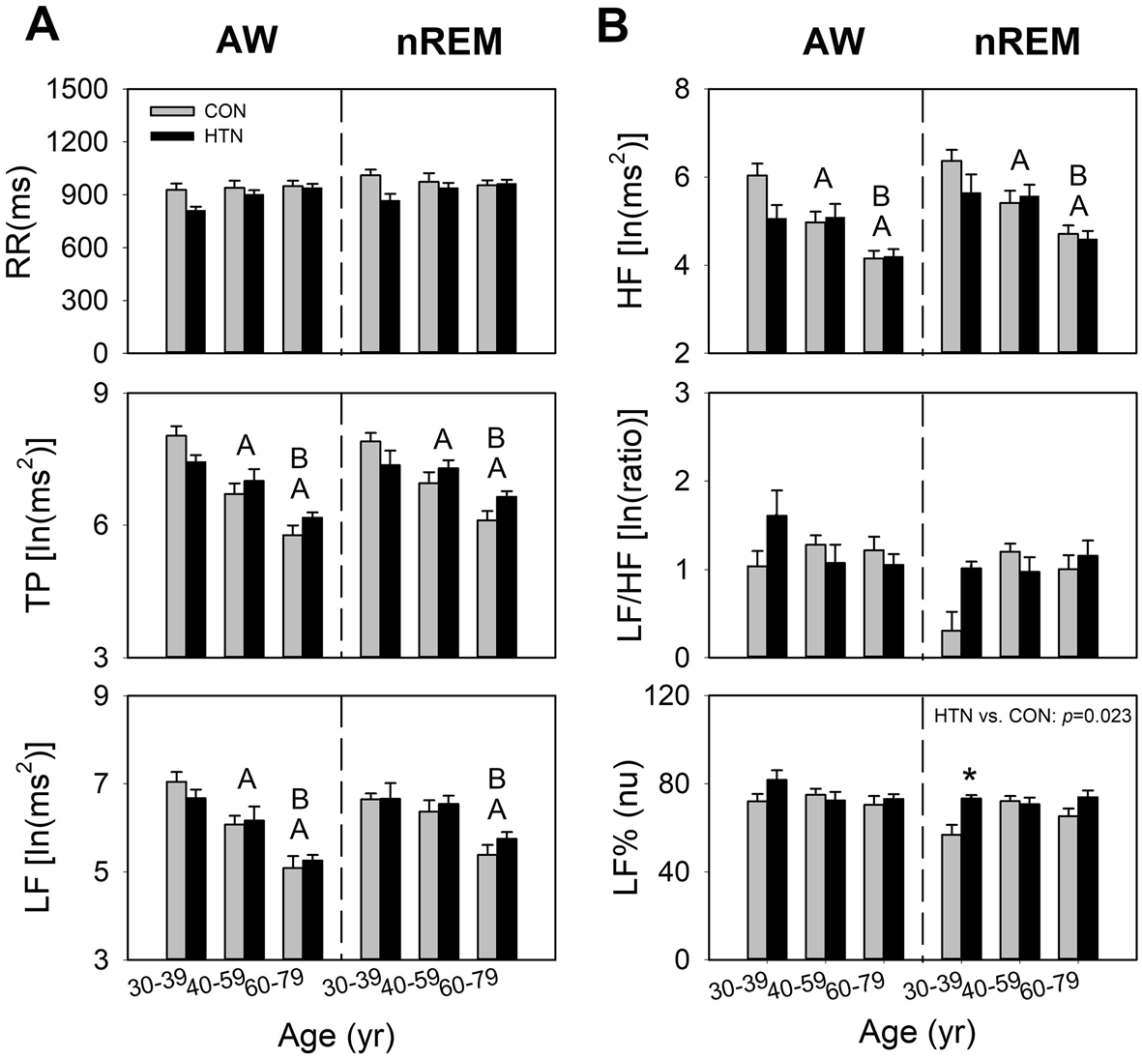
Influence of age and hypertension on HRV parameters in young, middle-aged, and older participants. For each group of samples (CON, HTN), each bar represents the average means of the parameters within the corresponding age range. *RR* R–R intervals, *TP* total power, *LF* low frequency, *HF* high frequency, *LF/HF* LF to HF ratio, *LF%* normalized LF, *AW* wakefulness, *nREM* nonrapid eye movement sleep, *CON* control group, *HTN* hypertension group. Data are presented as mean ± SEM, two-way ANOVA. *p < 0.05 HTN vs. CON. (**A**) *p* < 0.05 main effect of age, vs. 30–39; (**B**) *p* < 0.05 main effect of age, vs. 40–59. HTN vs. CON *p* < 0.05 main effect of HTN.

**Figure 2:**
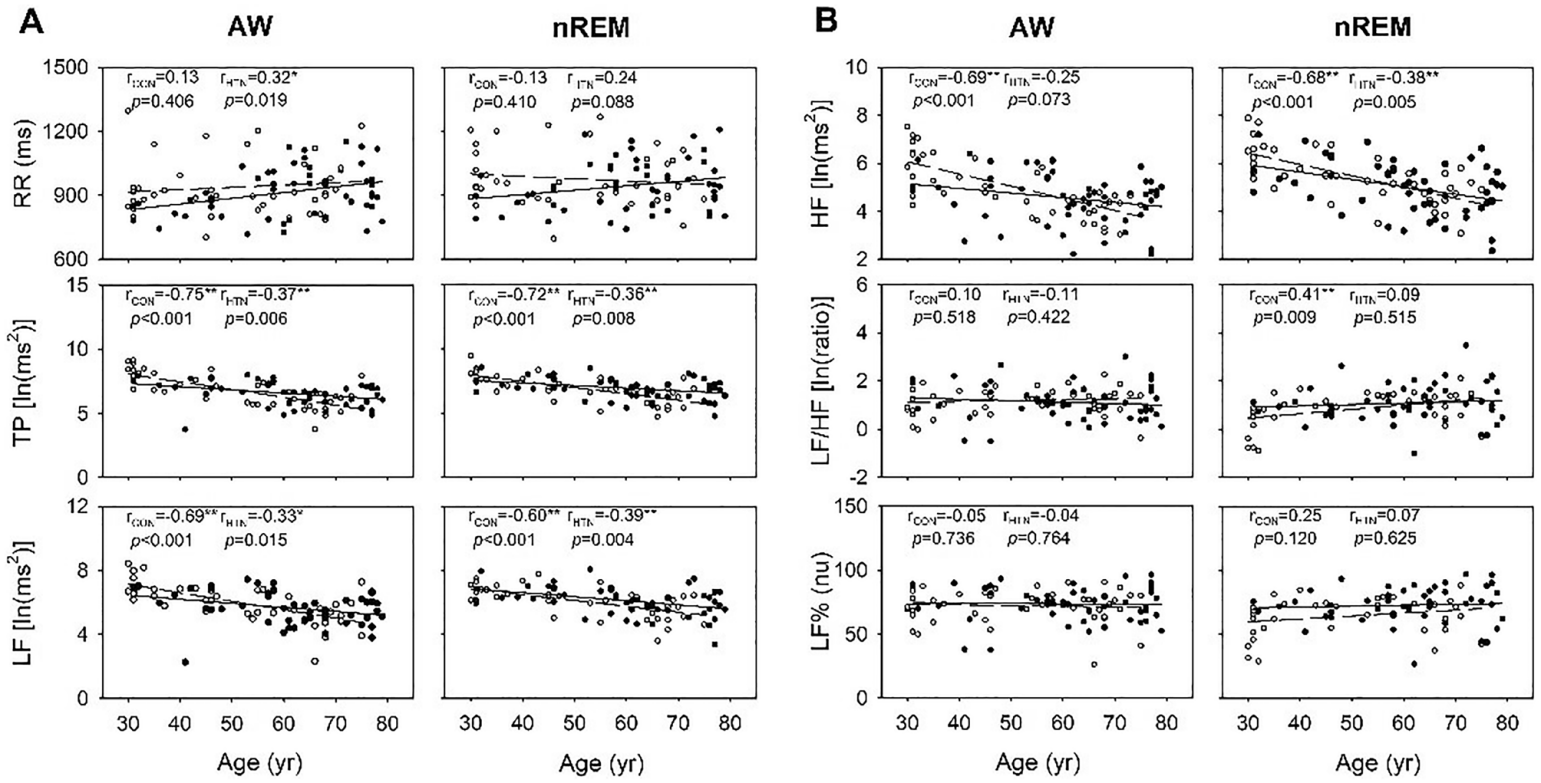
Two-dimensional scatter plots depicting the correlations between HRV parameters and age. *p < 0.05. *r*_*CON*_ correlation coefficient of the control group, *r*_*HTN*_ correlation coefficient of the hypertension group, *RR* R–R intervals, *TP* total power, *LF* low frequency, *HF* high frequency, *LF/HF* LF to HF ratio, *LF%* normalized LF, *AW* wakefulness, *nREM* nonrapid eye movement sleep.

The two-way ANOVA revealed a main effect of age on parasympathetic function–related cardiac parameters, including TP, LF, and HF, during both wakefulness and nREM (Table 2). The values of these parameters were the highest in the younger group, followed by the middle-aged and older groups (Fig. 1A,B). Although the age × hypertension interaction was nonsignificant, gradual age-dependent reductions were observed in TP, HF, and LF in the both groups. Consistently, TP, HF, and LF were found to be negatively correlated with age. During wakefulness, a strong correlation was observed in the control group and a weak to moderate correlation was observed in the hypertension group (respectively; TP, − 0.75 vs − 0.37 [*p* = 0.003]; LF, − 0.69 vs − 0.33 [*p* = 0.008]; HF, − 0.69 vs − 0.25 [*p* = 0.002]) and nREM (respectively; TP, − 0.72 vs − 0.36 [*p* = 0.007]; LF, − 0.60 vs − 0.39 [*p* = 0.098]; HF, − 0.68 vs − 0.38 [*p* = 0.024]; Fig. 2A,B). The correlation analysis also identified a medium relationship between LF/HF and age in the control group during nREM.

**Table 2:** Results of a two-way ANOVA test for the effects of hypertension and age on the HRV in young, middle-aged, and older participants.

Parameters	Source of variance	Wakefulness			nREM		
		F	*p*	Post hoc	F	*p*	Post hoc
RR	Hypertension	3.508	0.064		3.492	0.065	
	Age	2.087	0.130		0.133	0.875	
	Hypertension × age	1.042	0.357		2.008	0.140	
TP	Hypertension	0.024	0.876		0.357	0.552	
	Age	27.255	< 0.001	a > b; a > c; b > c	17.090	< 0.001	a > b; a > c; b > c
	Hypertension × age	2.050	0.135		2.630	0.078	
HF	Hypertension	1.434	0.234		0.995	0.321	
	Age	14.511	< 0.001	a > b; a > c; b > c	13.255	< 0.001	a > b; a > c; b > c
	Hypertension × age	1.884	0.158		1.018	0.366	
LF	Hypertension	0.037	0.848		0.930	0.337	
	Age	21.756	< 0.001	a > b; a > c; b > c	15.092	< 0.001	a > c; b > c
	Hypertension × age	0.491	0.614		0.298	0.743	
LF/HF	Hypertension	0.188	0.665		1.400	0.240	
	Age	0.437	0.647		1.969	0.146	
	Hypertension × age	1.973	0.145		1.937	0.150	
LF%	Hypertension	1.077	0.302		5.395	0.023	Hypertension > control
	Age	0.834	0.438		0.945	0.393	
	Hypertension × age	1.040	0.358		2.025	0.138	

a—individuals aged 30–39 years; b—individuals aged 40–59 years; c—individuals aged 60–79 years.

The two-way ANOVA revealed a significant main effect of hypertension on LF% during nREM, with an elevated LF% in the hypertension group (Table 2, Fig. 1B). Although the age × hypertension interaction was nonsignificant, the elevation in LF% occurred primarily in younger patients with hypertension (*p* = 0.004, vs control; independent *t* test).

### Effects of age and hypertension on vascular autonomic measures during sleep and wakefulness

**Figure 3:**
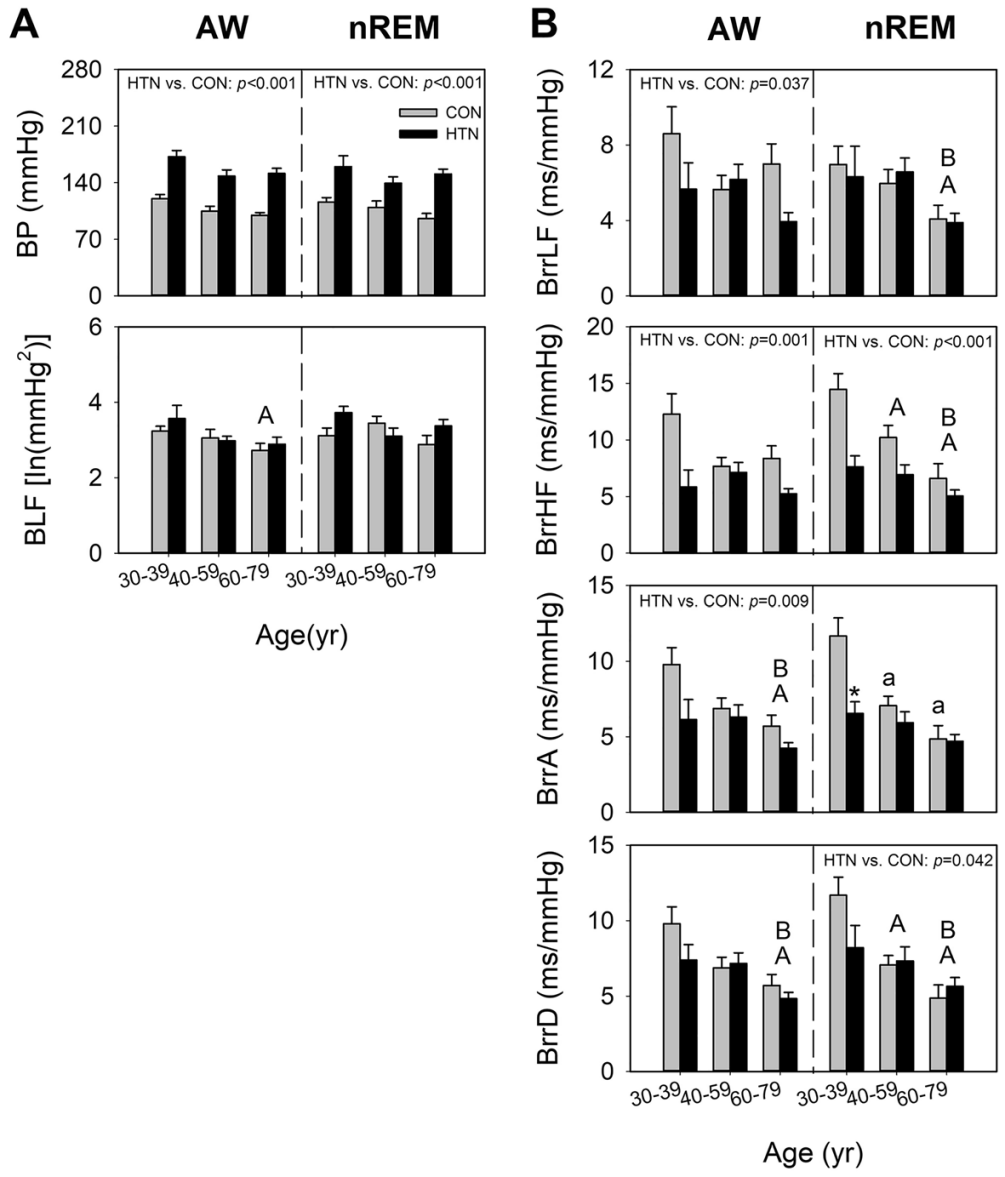
Effects of age and hypertension on arterial pressure variability/BRS parameters in young, middle-aged, and older men. Each bar in the graph represents the average values of the parameters within the respective age range for each group (HTN vs. CON). *ln* natural logarithm, *BP* blood pressure, *BLF* LF of arterial pressure variability, *BrrLF/BrrHF* magnitude of mean arterial pressure—R–R interval transfer function, *BrrA/BrrD* slope of mean arterial pressure—R–R interval linear regression, *AW* wakefulness, *nREM* nonrapid eye movement sleep, *CON* control group, *HTN* hypertension group. Data are presented in terms of the mean ± SEM values. (**A**) *p* < 0.05 main effect of age, vs. 30–39; (**B**) *p* < 0.05 main effect of age, vs. 40–59. a *p* < 0.05 vs. 30–39, least-significant difference post hoc test. *p < 0.05 vs. control of the same age group, independent-samples t test.

**Figure 4:**
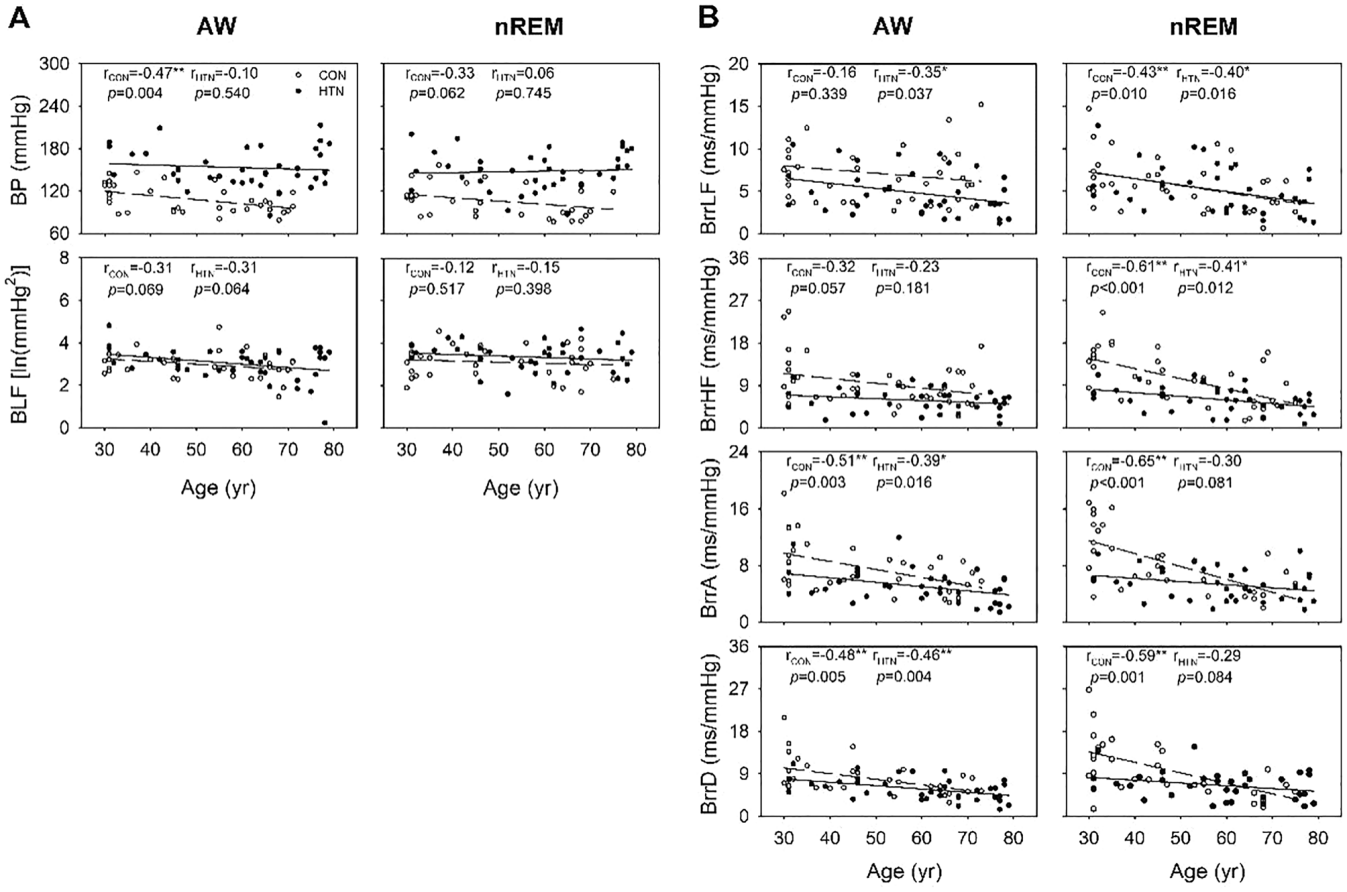
Two-dimensional scatter plots depicting the correlations between arterial pressure variability/BRS parameters and age. *p < 0.05. *r*_*CON*_ correlation coefficient of the control group, *r*_*HTN*_ correlation coefficient of the hypertension group, *ln* natural logarithm, *BP* blood pressure, *BLF* LF of arterial pressure variability, *BrrLF/BrrHF* magnitude of mean arterial pressure—R–R interval transfer function, *BrrA/BrrD* slope of mean arterial pressure—R–R interval linear regression, *AW* wakefulness, *nREM* nonrapid eye movement sleep.

The two-way ANOVA revealed a main effect of age on BrrLF, BrrHF, BrrA, BrrD, and BLF, with the effect being particularly more significant during nREM than during wakefulness (Table 3, Fig. 3B). During nREM, these values were highest in the younger group, followed by the middle-aged and older groups. During wakefulness, the effect of age was observed on BrrA, BrrD, and BLF, with the same trend of difference (Fig. 3A,B). In terms of BLF, the respective trends in age-related changes are similar in both groups during wakefulness (− 0.31 vs − 0.31, respectively) and sleep (− 0.12 vs − 0.15, respectively) (Fig. 4A). The correlation analysis revealed stronger correlations of age with BrrHF, BrrA, and BrrD in the control group than in the hypertension group (BrrHF: − 0.61 vs − 0.41, respectively [*p* = 0.132]; BrrA: − 0.65 vs − 0.30, respectively [*p* = 0.036]; BrrD: − 0.59 vs − 0.29, respectively [*p* = 0.072]), particularly during nREM (Fig. 4B). During wakefulness, the coefficient of the correlation between age and BrrA tended to be higher in the control group than in the hypertension group (− 0.51 vs − 0.39, respectively; *p* = 0.275). During nREM, a significant effect of the age × hypertension interaction was noted on BrrA (Table 3). In the younger group, BrrA was lower in patients with hypertension than in individuals without hypertension. However, no such difference was noted in the middle-aged or older group (Fig. 3B). Early reductions were also noted in BrrD and BrrHF in younger participants with hypertension (Fig. 3B). The small age-dependent changes in barosensitivity in patients with hypertension may reflect an early suppression of the baroreflex from a young age.

**Table 3:** Results of two-way ANOVA test for the effects of hypertension and age on the arterial pressure variability parameters in young, middle-aged, and older participants.

Parameters	Source of variance	Wakefulness			nREM		
		F	*p*	Post hoc	F	*p*	Post hoc
BP	Hypertension	70.414	< 0.001	Hypertension > control	44.639	< 0.001	Hypertension > control
	Age	4.464	0.015		1.902	0.158	
	Hypertension × age	0.248	0.781		1.574	0.215	
BLF	Hypertension	0.620	0.434		1.813	0.183	
	Age	3.606	0.033	a > c	0.854	0.431	
	Hypertension × age	0.379	0.686		2.515	0.089	
BrrLF	Hypertension	4.524	0.037	Control > hypertension	0.014	0.907	
	Age	1.238	0.296		6.680	0.002	a > c; b > c
	Hypertension × age	2.035	0.139		0.267	0.766	
BrrHF	Hypertension	11.949	0.001	Control > hypertension	17.338	< 0.001	Control > hypertension
	Age	1.752	0.181		10.513	< 0.001	a > b; a > c; b > c
	Hypertension × age	2.558	0.085		2.514	0.089	
BrrA	Hypertension	7.364	0.009	Control > hypertension	8.510	0.005	Control > hypertension
	Age	6.446	0.003	a > c; b > c	10.960	< 0.001	a > b; a > c; b > c
	Hypertension × age	1.447	0.243		3.814	0.028	
BrrD	Hypertension	3.798	0.056		4.325	0.042	Control > hypertension
	Age	8.492	0.001	a > c; b > c	7.812	0.001	a > b; a > c; b > c
	Hypertension × age	0.485	0.618		2.006	0.143	

a—individuals aged 30–39 years; b—individuals aged 40–59 years; c—individuals aged 60–79 years.

Hypertension significantly affected BP, BrrA, and BrrHF during both wakefulness and nREM (Table 3), BrrLF during wakefulness, and BrrD during nREM, with all values being lower in the hypertension group than in the control group (Table 3).

## Discussion

Our findings revealed that the age-dependent decreases in cardiac parasympathetic measures and BRS were less prominent in patients with hypertension than in individuals without hypertension, probably because of an early decrease in baroreflex at a young age. Patients with hypertension had higher cardiac sympathetic activity during nREM, with a more remarkable early decrease in BRS, than did individuals without hypertension. These findings suggest that autonomic disturbance, particularly impaired baroreflex, plays a major role in younger patients with hypertension and also indicate that nREM is an essential yet often overlooked time window for gaining insights into this mechanism.

### Autonomic functioning in hypertension

Excessive sympathetic nervous system activity and reduced parasympathetic activity, in addition to decreased BRS, have been associated with hypertension. Consistent with our findings, a study demonstrated that patients with essential hypertension who were aged approximately 50 years exhibited mean HR and LF power during rest that were similar to those of age-matched individuals without hypertension^32^. In patients with essential hypertension, heart rate control by baroreceptors is reduced^33^. Furthermore, the occurrence of spontaneous periodic breathing is associated with increased sympathetic responsiveness and reduced resting cardiac BRS^34^. However, we did not observe any significant difference in vascular sympathetic activity between patients with hypertension and those without hypertension, likely because of the use of different measurement techniques. Nonetheless, our study revealed a prominent difference in BRS index variations between patients with hypertension and individuals without hypertension.

### Aging and autonomic functioning in patients with hypertension

The sympathetic and parasympathetic branches of cardiac ANS exert the effects in the human myocardium through activation of specific G protein-coupled receptors, namely the adrenergic and muscarinic cholinergic receptors, respectively^35^. Dysregulations in the cardiac ANS are linked to cardiovascular diseases and aging^35,36^. The general effects of aging on the ANS are mediated through a reduction of cardiovagal BRS, the maintenance or an increase of sympathetic activity, and aberrant β-adrenergic signaling^33,36^. In individuals without hypertension, a twofold increase was noted in the LF/HF ratio with increasing age^37,38^. Our findings pertaining to individuals without hypertension are consistent with those of other studies^8,9^. By contrast, in patients with hypertension, no variation is observed in the LF/HF ratio^37,38^ or BP variability^39^ with increasing age, probably because the HRV characteristics of younger patients with hypertension are similar to those of older patients with hypertension^37^. Because of the limited sample size of our study, the reduction in parasympathetic activity in younger patients with hypertension did not reach statistical significance. Our results revealed that the sympathovagal imbalance in hypertension was the most prominent in younger patients. In middle-aged and older patients with hypertension, sympathovagal imbalance did not aggravate with increasing age. Therefore, the mechanisms underlying hypertension may vary across different age groups with hypertension.

### Pathologic mechanisms of hypertension in different age groups

Pathophysiology of essential hypertension may differ between older and young patients^40^. During the initial phases of essential hypertension, an increase in observed in sympathetic drive^41^. The overflow of norepinephrine to the heart and kidney is increased in patients with essential hypertension^42^, particularly in patients aged < 40 years^43^. In younger patients, heightened renal sympathetic nerve activity correlates with elevated levels of renal renin secretion and arterial plasma renin activity, whereas in older patients, the increase in sympathetic nerve activity and norepinephrine release may vary^44^. In an animal study, severe, self-sustaining salt hypertension in young Dahl rats was associated with enhanced sympathetic nervous system activity, reduced nitric oxide levels, weakened baroreflex response, and increased residual BP, all indicating extensive remodeling of resistance blood vessels; these findings differed from those observed in adult Dahl rats^45^.

As age advances, the reduced vagal output leads to a pro-inflammatory state associated with endothelial dysfunction^46^. Moreover, there is a decline in baroreceptor function, reflex efficiency, and renal excretory capacity, potentially significantly promoting the development of hypertension^47,48^. However, age-related alterations in sympathetic nervous system and parasympathetic nervous system don’t progress linearly, indicating complex interrelationships among factors^44^. Therefore, both aging and autonomic imbalance contribute to the pathogenesis of hypertension in older individuals, with autonomic imbalance playing a more significant role in younger patients with hypertension^47,49^.

### Importance of sleep

The ANS exhibits substantial dynamic variations across sleep–wake cycles. Under physiological condition, sympathetic activity, BP, and HR are lower and parasympathetic activity is higher during nREM than during wakefulness^50^. Spontaneous hypertensive rats had lower HF during wakefulness than do normotensive rats; however, the LF/HF ratio and LF% were similar between the rat groups. During paradoxical sleep, spontaneously hypertensive rats had significantly lower RR, higher LF/HF ratio, higher LF%, and lower HF than did Wistar–Kyoto rats^15,23^. However, most studies involving patients with hypertension assessed autonomic function only during wakefulness. A study in which 24-h data were assessed revealed considerable differences in HRV parameters patients with hypertension and individuals with hypertension during nighttime: LF was lower and HF was higher in individuals without hypertension, whereas the LF/HF ratio was higher in patients with hypertension^51^. However, the aforementioned study was confined to individuals aged approximately 40 years; moreover, it did not truly differentiate between wake and sleep states. Sleep is a state with fewer external disturbances, which allows for a precise assessment of the cardiovascular state. In our study, during the nREM stage, where a physiological decrease in sympathetic activity is expected, we noted a significant increase in LF% among patients with hypertension.

### Limitations

Our study has some limitations. Notwithstanding its appeal, our method possesses notable constraints, particularly when used for the quantitative and specific assessment of cardiac sympathetic activity versus parasympathetic activity. Moreover, its focus is limited to heart rate regulation by the sympathetic system, precluding a broader understanding. Similar reservations apply to the power spectral analysis of arterial pressure as an indicator of generalized sympathetic activity. Because of the significant regional differences in the characteristics, control, and function of the sympathetic nervous system as well as the existence of multiple levels of this nervous system, no single method is adequate for the overall assessment of sympathetic nerve activity or tone. In essence, our assessment of the ANS remains incomplete. Subsequent investigations could encompass a broader array, incorporating assessments such as sympathetic nerve activity, tilt table testing, Valsalva maneuver, serum catecholamine levels, time-domain analysis of HRV, and sympathetic skin response, among others. Moreover, although we divided the participants into three age groups, the actual onset time of hypertension might have varied considerably within each age group. These limitations should be considered when interpreting the pathological mechanisms. Future studies should comprehensively explore this topic in an integrated manner. Additionally, our research specifically focused on men of a single ethnicity, so one should exercise caution when generalizing our findings to populations beyond the scope of our study. Finally, we could not eliminate the potential influence of medications targeting autonomic cardiac function, such as antihypertensive drugs.

## Conclusions

Monitoring the ANS function during wakefulness and nREM in patients with essential hypertension improves our understanding of the patient population-specific dynamic changes in nerve activity during these stages, which may advance cardiovascular research. Assessing the ANS dynamics, particularly during nREM, can help elucidate the inherent influences on hypertension. The etiology of essential hypertension is multifactorial. This study highlights the significance of neural factors, particularly those involved in cardiovascular neural regulation during sleep, as key factors in essential hypertension. This phenomenon is apparent even in younger patients. The present study provides insights into the essential hypertension-related alterations in autonomic neural control. Our findings may guide the selection of appropriate medications and timely management of essential hypertension in younger patients.

### Supplementary Information

Supplementary Figure S1.

## Data Availability

All data generated or analysed during this study are included in this published article (and its Supplementary Information files).
