# Supplementary material for: Role of the autonomic nervous system in young, middle-aged, and older individuals with essential hypertension and sleep-related changes in neurocardiac regulation
Source: Sci Rep. 2023 Dec 18;13:22623. doi: 10.1038/s41598-023-49649-2 (PMC10730708; doi:10.1038/s41598-023-49649-2)
Supplement: Supplementary file 1 — Supplementary Figure S1. [file 41598_2023_49649_MOESM1_ESM.docx]

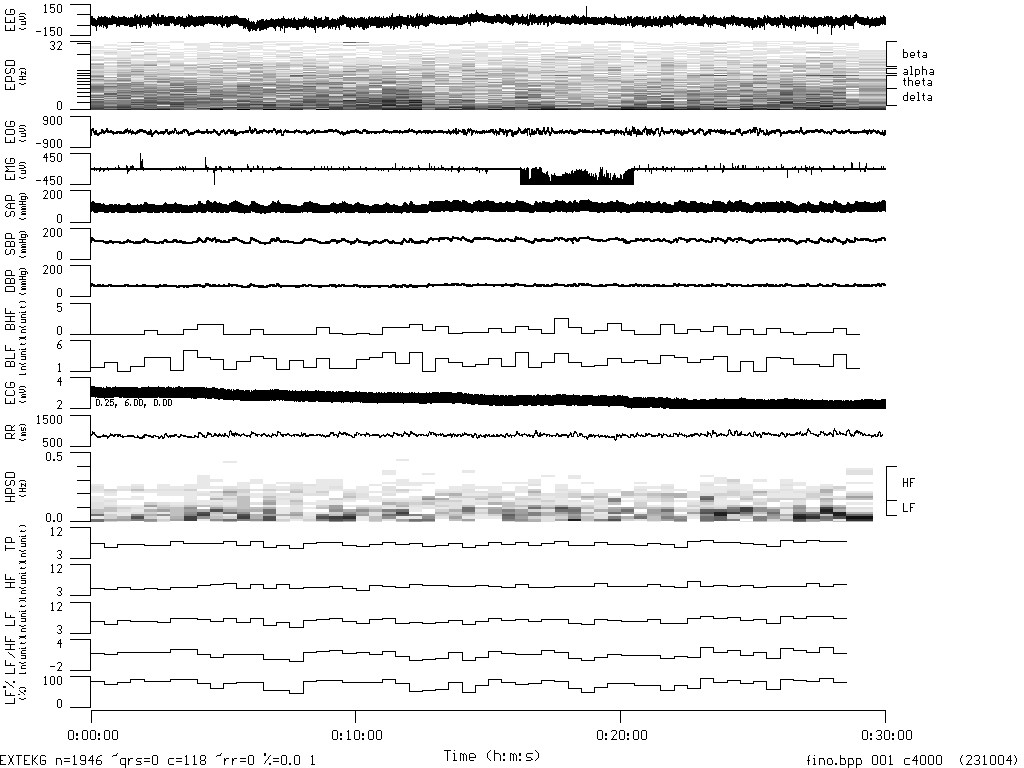


**Fig. S1. Intranap PSG, BP, and HRV recordings of a patient.**

Throughout the nap experiment, PSG data, BP, and HRV were measured simultaneously. In addition to continuous BP monitoring, the continuous variations of the EEG and the three-dimensional power spectral density (EPSD) of the EEG were monitored. The temporal variations in the power of spectral components were also recorded. Eye movement graph (EOG), electromyogram (EMG), and RR as well as the corresponding three-dimensional power spectral density (HPSD) of RR, quantified values of HF power and LF power in the spectrum, and the LF/HF ratio were also monitored.

PSG, polysomnography; BP, blood pressure; HRV, heart rate variability.
